# Diazoxide as a bridging therapy in methadone-induced hypoglycemia– a case report and review of the literature

**DOI:** 10.1186/s40842-025-00220-0

**Published:** 2025-04-25

**Authors:** Katherine I. Wolf, Sophia Hemmrich Sinha, Shafaq Khairi, Eric D. Buras

**Affiliations:** Department of Internal Medicine, Division of Metabolism, Endocrinology, & Diabetes. Michigan Medicine, NCRC B020, 2800 Plymouth Rd, Ann Arbor, MI 48109-2800 USA

**Keywords:** Hyperinsulinemic, Hypoglycemia, Methadone, Tramadol, Diazoxide

## Abstract

**Background:**

The objective of this report is to describe the successful use of diazoxide as a bridging therapy for a patient with opioid-induced hypoglycemia who was unable to immediately discontinue methadone treatment.

**Case presentation:**

A 41-year-old male presented for an unrelated planned surgical procedure and was found to have symptomatic hyperinsulinemic hypoglycemia resulting from high-dose methadone prescribed for chronic pain in the setting of end stage renal disease requiring hemodialysis. Diazoxide was successfully used as a bridging therapy until he ultimately decided to discontinue methadone following a multidisciplinary discussion with endocrinology, internal medicine, and an addiction consultation team. During a 10-day admission for safety and observation of withdrawal, he was weaned from methadone and transitioned to buccal buprenorphine and eventually sub-lingual buprenorphine/naloxone. After methadone was decreased and then discontinued, he was able to discontinue dextrose infusion and eventually diazoxide. Several months after discharge, he reported fasting blood glucose levels between 110 and 130 mg/dL (6.11–7.22 mmol/L; reference range 75–115 mg/dL; 4.16–6.38 mmol/L) without symptoms of hypoglycemia.

**Conclusions:**

While diazoxide has been used as bridging therapy for insulinoma patients awaiting surgery, this report highlights the utility of the non-diuretic benzothiadiazine in opioid-induced hyperinsulinemic hypoglycemia. Patients with hyperinsulinemic hypoglycemia on high doses of tramadol or methadone should be transitioned off the offending agent; however, diazoxide is an effective, alternative option when the transition is not feasible or needs to be delayed.

## Background

Hypoglycemia in the setting of Whipple’s triad– signs or symptoms consistent with hypoglycemia, a low plasma glucose concentration, and resolution of these signs or symptoms when glucose is raised– in the adult, non-diabetic population requires a thorough evaluation. In 2009, the Endocrine Society published a clinical practice guideline recommending patients undergo evaluation to distinguish endogenous from exogenous insulin production while reviewing history to determine if drugs, critical illness, or other hormone deficiencies could be contributing to their current presentation [1]. As of the early 2000s, reports estimated that approximately 20% of hospital admissions attributed to adverse drug reactions were a result of hypoglycemia [2], which is perhaps unsurprising given that more than 160 agents are currently associated with drug-induced hypoglycemia [3]. Opioids, particularly methadone and tramadol, are known to result in drug-induced hyperinsulinemic hypoglycemia (DHH) [4]. To date, eight case reports from 2015 to 2024 and one letter to the editor have reported methadone induced hyperinsulinemic hypoglycemia with inappropriately elevated insulin and c-peptide levels [5–13]. Here, we report the successful use of diazoxide as a bridging therapy for methadone induced hyperinsulinemic hypoglycemia. While the definitive treatment required transition off methadone, this report highlights the effectiveness of diazoxide as an alternative agent.

## Case presentation

A 41-year-old male with a history of end stage renal disease (ESRD) on intermittent hemodialysis (iHD) in the setting of lupus nephritis, heart failure with reduced ejection fraction and chronic pain (on methadone) was admitted for planned nephrectomy for an incidentally found 3.8 cm renal mass. Post-operatively, he was persistently hypoglycemic with point-of-care (POC) blood glucose levels (BG) ranging from 50 to 70 mg/dL corresponding with lightheadedness and dizziness that resolved with food.

Chart review of an outside hospital admission one year prior identified multiple POC BG checks between 40 and 60 mg/dL (2.22–3.33 mmol/L; reference range (RR) 75–115 mg/dL; 4.16–6.38 mmol/L); as well as a value of 62 mg/dL (3.44 mmol/L) on blood draw that was reportedly not associated with symptoms. Further workup performed during this encounter was unavailable in the medical record. The patient reported being told to monitor his BG but did not have a glucometer. He also reported intermittent weakness and pre-syncope over the next year that resolved with eating—leading him to snack consistently throughout the day—however, he was not awakened by hypoglycemia symptoms. He denied any weight loss, skin darkening, and prior history of diabetes or bariatric surgery. He further denied any access to insulin or oral hypoglycemic agents.

Review of prescription drug use was notable for various doses of prednisone ranging from 5 to 60 mg daily over the last 20-years; however, he self-discontinued steroids more than 12-months prior to admission. Given his ESRD, he was not using nonsteroidal anti-inflammatory drugs (NSAIDs); and he had not recently taken antibiotics. His long-term, stable methadone was dosed at 175 mg daily. He had no family history of endocrine-related tumors.

Throughout admission, he was afebrile with a normal heart rate and systolic blood pressures ranging from 90 to 100 mmHg. On physical examination, he was slender, appeared older than stated age, and was fatigued without rash or hyperpigmentation. His abdomen was soft and diffusely tender—attributable to his recent procedure.

Adrenocorticotropic hormone (ACTH) stimulation was performed given concern for secondary adrenal insufficiency after abrupt discontinuation of decades-long glucocorticoid dosing; however, cortisol was appropriately stimulated, reaching 32.3 ug/dL (891.16 nmol/L; RR 5–23 ug/dL; 137.95-634.57 nmol/L) 30 min after ACTH administration—baseline 11.4 ug/dL (314.53 nmol/L). He subsequently underwent a 72-hour fast, during which dextrose-containing fluids were held. Five hours after initiation of the fast, POC BG declined to 46 mg/dL (2.55 mmol/L) with corresponding value of 56 mg/dL (3.11 mmol/L) on blood draw. Hypoglycemic agent screen (chlorpropamide, glimepiride, glipizide, glyburide, nateglinide, pioglitazone, repaglinide, rosiglitazone, tolazamide, and tolbutamide), thyroid stimulating hormone, and insulin antibody testing were negative, while insulin and c-peptide were inappropriately elevated to 20.2 uU/mL (144.94 pmol/L; RR 1.0–21.0 uU/mL; 43.05–172.2 pmol/L) and 8.1 ng/mL (2.67 nmol/L; RR 1.0-5.2 ng/mL; 0.17–1.32 nmol/L), respectively. Computed tomography (CT) of the pancreas was unremarkable, aside from expected post-operative changes given recent nephrectomy.

With continued symptomatic hypoglycemia, the patient started diazoxide 50 mg twice daily (BID) and a tablespoon of cornstarch at night (qHS) with plan to evaluate for insulinoma by endoscopic ultrasound (EUS) as an outpatient. Despite discussions on potential contribution of methadone to his presentation, the patient deferred its discontinuation. On the above regimen, he was euglycemic for more than 48-hours without dextrose-containing fluids and subsequently discharged.

Two months later, he again presented to the emergency department complaining of worsening abdominal pain, nausea, and vomiting; and was found to have POC BG < 50 mg/dL on multiple checks. Notably, frequent bouts of emesis over the prior week had prevented adequate intake of food and medications (including diazoxide); and pre-syncopal symptoms had re-emerged. After a multidisciplinary meeting between Endocrinology, the Addiction Consult Team (ACT), Gastroenterology, and General Internal Medicine, EUS was deferred until less-invasive investigations into the etiologies of hyperinsulinemic hyperinsulinemia had been pursued —including investigating the role of opioids. The ACT requested approval for a minimum 10-day admission for safety and observation where they devised an individualized treatment plan to transition from methadone to buprenorphine/naloxone (Table 1). The patient agreed and signed a contract with ACT stating that he understood the increased risk for withdrawal-like symptoms during this period and that his pain may worsen before improvement. Multiple supportive medications, including ketamine, clonidine, and hydroxyzine were available as needed along with a standard regimen for nausea and diarrhea.

**Table 1:** Individualized buprenorphine initiation plan

Day	Buprenorphine dose	Methadone dose	Supportive Medications
4	Buprenorphine 150 mcg buccal q6H	Methadone 175 mg PO daily	Ketamine 10 mg PO TID; monitor weekly LFTs Clonidine 0.1 mg PO TID PRN; HOLD for SBP < 100, HR < 60 Hydroxyzine 50 mg PO q6H Loperamide 4 mg PO PRN followed by 2 mg q4H PRN. MAX 16 mg/day Prochlorperazine 10 mg PO q6H PRN NO NSAID– patient with history of stomach ulcers and ESRD on iHD
5	Buprenorphine 300 mcg buccal q6H	Methadone 175 mg PO daily	
6	Buprenorphine 450 mcg buccal q6H	Methadone 175 mg PO daily	
7	Buprenorphine 600 mcg buccal q6H	Methadone 175 mg PO daily	
8	Buprenorphine 900 mcg buccal q6H	Methadone 175 mg PO daily	
9	Buprenorphine/Naloxone 2-0.5 mg SL TID	Methadone 80 mg PO daily	
10	Buprenorphine/Naloxone 4 − 1 mg SL TID	Methadone 80 mg PO daily	
11	Buprenorphine/Naloxone 16 − 4 mg SL x1 followed by Buprenorphine/Naloxone 8 − 2 mg SL TID	NONE	
12	Buprenorphine/Naloxone 8 − 2 mg SL TID	NONE	

Abbreviations: ESRD (end stage renal disease), HR (heart rate), iHD (intermittent hemodialysis). LFTs (liver function tests), mcg (micrograms), mg (milligrams), NSAID (nonsteroidal anti-inflammatory drug), PO (by mouth), PRN (as needed), q4H (every four hours), q6H (every six hours), SBP (systolic blood pressure), SL (sublingual), TID (three times daily)

**Fig. 1:**
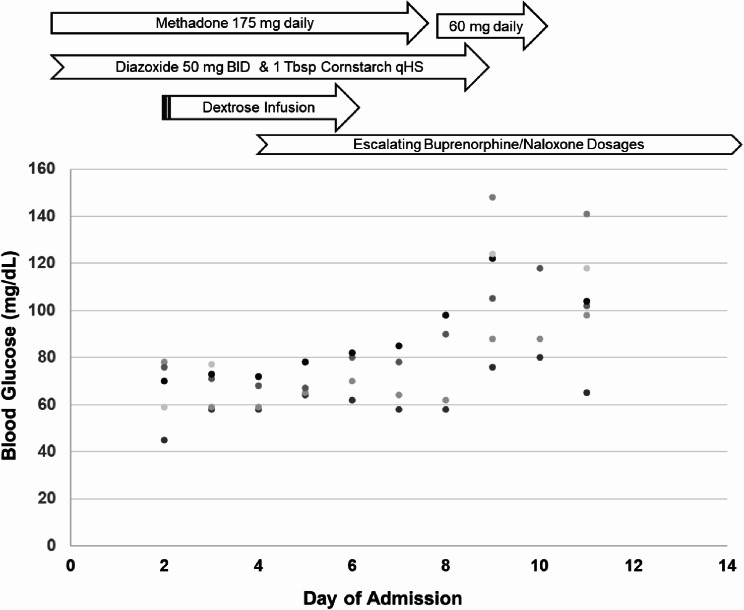
Blood Glucose Levels During Transition from Methadone to Buprenorphine/ Naloxone. Point of care blood glucose values (y-axis) plotted throughout the hospital admission (x-axis). Prior to admission, the patient was taking home dose of methadone, diazoxide, and cornstarch. During admission, he was started on dextrose infusion due to poorly controlled hypoglycemia. Following escalating doses of buprenorphine/naloxone and discontinuation of methadone, he transitioned off dextrose infusion along with his bridging therapies of diazoxide and cornstarch

After methadone was halved on day six of the treatment plan (Fig. 1), the patient was able to wean from diazoxide and cornstarch. On day seven, he transitioned off methadone completely and was eventually discharged several days later without hypoglycemia for 48 h despite taking no agents to increase BG. Several months following the transition, the patient reported he routinely checks fasting POC BG, with values ranging from 110 to 130 mg/dL (6.11–7.22 mmol/L). His pre-syncopal symptoms had resolved. Moreover, his pain was well-controlled, though he disliked the taste of the buprenorphine/naloxone film. He also noted that finding an outpatient provider had proved more challenging than attending his methadone clinic, but he was working with the ACT find a solution.

## Discussion and conclusions

While the patient initially re-presented to the hospital with acute hypoglycemia two-months post initial discharge, we hypothesize his gastrointestinal upset was the result of a viral illness and unrelated to DHH. Had frequent emesis not prevented him from adequate intake of both food and his medications, we suspect diazoxide would have continued to relieve his symptomatic hypoglycemia.

Including our case, there have now been ten reports detailing DHH in the setting of opioids (5 male, 5 females; 8 adult, 2 children, age range 11-months to 70-years-old [5–13]). Among these, six reported c-peptide and insulin levels, which were inappropriately elevated in all cases except one^6,8–10−11^. In 3/ 7 previously reported adult cases, hypoglycemia resolved after transition from methadone to bupropion [10] or buprenorphine/naloxone^8–9^. One patient was found unresponsive in a rehabilitation facility with a BG < 40 and later pronounced dead. “Adverse effect of methadone” was listed as the cause of death; and it was hypothesized that prolonged hypoglycemia had caused sudden cardiac death [7]. In two cases, patients deferred transition to alternative medications and were discharged on reduced methadone doses (11–12)—and, in one case related to intentional massive methadone overdose, the patient was started on naloxone infusion [13]. Including our own case, ESRD was present in four of the eight adult cases and may predispose methadone-induced hypoglycemia (8–9, 11). Of the two pediatric cases, one resulted from accidental exposure; and hypoglycemia resolved without recurrence following naloxone administration [6]. The other involved a patient subjected to rapid methadone dose escalation for cancer-related pain, in whom BG also normalized following dose reduction [5]. Finally, a retrospective chart review of opioid-tolerant patients with cancer receiving methadone in a single tertiary care center found that 19% experienced hypoglycemia following a near doubling of their methadone doses within two days of symptom onset—highlighting the need for close BG monitoring during rapid titration [14].

The proposed molecular mechanism underlying methadone-induced hyperinsulinemic hypoglycemia is the induction of insulin release resulting from direct action of methadone on mu opioid receptors of the b-cell [2]. An alternative hypothesis, suggested based on rodent, involves the known serotonergic effects of methadone (and tramadol) contributing to increased insulin production [2, 4]. Other opioids—including morphine, oxycodone, and fentanyl—have been demonstrated *not* to impact BG in mouse models, indicating that hyperinsulinemic hypoglycemia is not a general property of opioids [4, 15–17]. Furthermore, one of the aforementioned studies demonstrated that BG significantly decreased from pre-drug baseline with methadone doses greater than 15 mg/kg^4^.

With elevated insulin and c-peptide levels, methadone-induced hyperinsulinemic hypoglycemia may mimic an insulin-secreting islet cell tumor. Definitive treatment is to transition off therapy, but given the dose-dependency of hypoglycemia, lowering methadone dose may be sufficient to relive symptoms. Here, we have demonstrated diazoxide to be effective in relieving Whipple’s triad and restoring measured glycemic values to within normal limits. Further studies are needed to determine if diazoxide is a successful, long-term therapy for patients unable to transition from methadone, or if octreotide (which has proven beneficial in multiple other DHH) represents another viable bridging therapy.

## Data Availability

No datasets were generated or analysed during the current study.

## Abbreviations

ACT: Addiction consult team
ACTH: Adrenocorticotropic hormone
BID: Twice daily
BG: Blood glucose
CT: Computed tomography
DHH: Drug-induced hyperinsulinemic hypoglycemia
ESRD: End stage renal disease
EUS: Endoscopic ultrasound
iHD: Intermittent hemodialysis
NSAIDs: Nonsteroidal anti-inflammatory drugs
POC: Point-of-care
qHS: At night
RR: Reference range
